# Natural Products in Cancer Therapy: Past, Present and Future

**DOI:** 10.1007/s13659-020-00293-7

**Published:** 2021-01-03

**Authors:** Min Huang, Jin-Jian Lu, Jian Ding

**Affiliations:** 1 State Key Laboratory of Drug Research, Shanghai Institute of Materia Medica, Chinese Academy of Sciences, Shanghai, China; 2 University of Chinese Academy of Sciences, Beijing, China; 3 State Key Laboratory of Quality Research in Chinese Medicine, Institute of Chinese Medical Sciences, University of Macau, Macao, China

**Keywords:** Natural products, Cancer therapy, Drug discovery, Antibody–drug conjugates, Combination therapy

## Abstract

Natural products, with remarkable chemical diversity, have been extensively investigated for their anticancer potential for more than a half-century. The collective efforts of the community have achieved the tremendous advancements, bringing natural products to clinical use and discovering new therapeutic opportunities, yet the challenges remain ahead. With remarkable changes in the landscape of cancer therapy and growing role of cutting-edge technologies, we may have come to a crossroads to revisit the strategies to understand nature products and to explore their therapeutic utility. This review summarizes the key advancements in nature product-centered cancer research and calls for the implementation of systematic approaches, new pharmacological models, and exploration of emerging directions to revitalize natural products search in cancer therapy.

Given the remarkable chemical diversity in nature, natural products are considered as a rich reservoir of bioactive compounds with therapeutic potentials. In the past decades, tremendous efforts have been made to isolate novel natural products, from microbes, plants, and other living organisms, to assess their anticancer properties and to explore the mechanism of action. These endeavors had led to the discovery of a panel of anti-cancer drugs. It is estimated that between 1981 and 2019, approximately 25% of all newly approved anti-cancer drugs were related to natural products [1, 2]. Meanwhile, countless compounds with anticancer potentials or unique structural advantages in probing druggable modalities have been reported.

Regardless of these achievements, developing bioactive natural products into drugs has remained challenging, in part because of the difficulty in large-scale isolation, mechanistic understanding and pharmaceutical development. As a consequence, major pharmaceutical companies worldwide have reduced or even eliminated their efforts in natural products for drug discovery, and relied primarily on large libraries of chemically synthesized compounds or biologics instead. Recently, with the explosive growth of our knowledge in cancer therapy and innovative technologies, it becomes possible to overcome hurdles in improving the efficiency in drug discovery, revealing the direct targets of natural products, and resolving the complexity of the multi-faceted pharmacological effects. This review highlights the conceptual and technological advancement in natural products research, which hopes to provide insights of how to launch an effort to rediscover natural products and revitalize anticancer drug discovery.

## Natural Products in Clinical Cancer Treatment: The Glory in the History

Natural products have marked the history of anticancer drug discovery. A number of widely-used anticancer therapeutics originate from natural sources, such as irinotecan, vincristine, etoposide and paclitaxel from plants, actinomycin D and mitomycin C from bacteria as well as marine-derived bleomycin. Some of these compounds are still the mainstay of cancer therapy and will continue to play a pivotal role in the foreseeable future. Among them, camptothecin and taxol are undoubtedly the two most successful examples, both of which were identified between 1950 and 1960s in a campaign initiated by National Cancer Institute (NCI) to discover therapeutic values of natural products [3, 4]. Meanwhile, Chinese scientists made significant contributions to bringing arsenic trioxide, an old remedy in Traditional Chinese Medicine (TCM), to standard-care of acute promyelocytic leukemia (APL) [5].

### Camptothecin and Taxol

The anticancer activity of camptothecin, isolated from wood and bark of *Camptotheca acuminata*, was initially noted in the early 1960s, yet its application as an anticancer agent languished for almost 20 years until its mode of action was uncovered [6, 7]. Camptothecin is able to specifically trap topoisomerase I, an enzyme critically involved in both DNA replication and transcription processes, and form topoisomerase-DNA complexes. These complexes could cause severe genomic stress when collide with the ongoing DNA replication fork or transcription machinery, leading to cell death [8]. This unique mode of action rekindled interest in developing camptothecin analogs, with aims to improve solubility, reduce toxicity and retain anticancer activity. In the mid-1990s, two camptothecin analogs, topotecan and irinotecan, received Food and Drug Administration (FDA) approval for treating various types of cancer including ovarian, lung, breast and colon cancers, and 10-hydroxycamptothecin, with reduced toxicity compared with camptothecin, has been used against hepatoma, colon cancer and bladder cancer in China since 1970s.

The story of taxol (paclitaxel) is not much different, but with more hurdles, representing a typical journey of natural products to reach bedside. The first challenge came from the compound supply, as often occurred to natural products. Taxol was originally isolated from bark of *Taxus brevifolia*, which is a finite resource and only yields very small amount of the compound. The adequate quantity for therapeutic use was resolved by a commercially feasible semi-synthetic procedure, starting from 10-DAB that could come from a renewable plant resource. The complex chemical structure, as the next hurdle, was not resolved until 1971, with the collective assistance of mass spectrometry, X-ray crystallography and NMR spectroscopy, an approach quite common today but then still in their infancy. The mechanism of action of taxol was reported in 1979. Taxol was found to bind microtubules and cause dysfunction in microtubules dynamics, resulting mitotic catastrophe of cancer cells. The last obstacle came from the poor solubility, which was solved by a special formulation made of castor oil, marketed as Cremophor EL, finally paving the way for taxol to proceed to clinical trials. In December 1992, more than twenty years after initial report of its isolation and structure, FDA granted its approval for treating refractory ovarian cancer [3, 4, 7, 9].

Even today, the legacy of camptothecin and taxol is not vanishing. Follow-up work yielded a series of new camptothecin derivatives with improved drug properties and some of them had proceeded to clinical trials. Chimmitecan, a camptothecin derivative developed by scientists at Shanghai Institute of Materia Medica, Chinese Academy of Sciences, is undergoing phase II trial in China [8, 10]. Utilizing an albumin-bound nanoparticle (nab) technology, paclitaxel was able to circumvent the severe toxicities caused by the formulation and concentrate in tumors [11, 12]. Nab-paclitaxel has been approved in 2005 for the treatment of metastatic breast cancer, followed by indications in pancreatic cancer and non-small cell lung cancer.

### Arsenic Trioxide

Arsenic has been an old remedy in both western and Chinese traditional medicines for centuries. Beginning from 1970s, arsenic containing drugs have been used for treating APL in China, in the format of a preparation containing arsenic and a trace amount of mercury chloride (known as Ailing-1), which opened a prologue of treating APL with arsenic drugs [13–16]. The clinical data using the pure form of arsenic trioxide was reported in mid-1990s [17]. To date, clinical trials both from China and western countries have consolidated the remarkable benefit of arsenic trioxide in APL patients [18, 19]. Ever since, APL has become a highly curable disease. The outcome of APL patients was revolutionized by regimens combining retinoic acid and arsenic trioxide, reach a stunning 90% cure rate [20]. In-depth mechanistic studies afterwards reveal that arsenic trioxide exhibits a mechanism of action by degrading PML-RARα fusion protein, the oncogenic driver of APL. Arsenic trioxide targets the PML moiety of PML-RARα and specifically induces a SUMO-dependent degradation via the ubiquitination-proteasome system [21–25].

A nearly 30-year-long journey of arsenic trioxide illustrates how joint efforts from both clinicians and basic researchers have transformed this primitive and mysterious “poison” into a modernized targeted therapy with well-understood mechanism of action. Moreover, as often occurring in natural products, arsenic trioxide’s unique chemical property enables an advantage to probe the molecular basis of APL, yielding a PML-RARα degradation strategy for APL treatment that otherwise not have been able to reveal.

## Recent Advancement of Natural Products in Anticancer Drug Discovery

Most of naturally-derived drugs launched around 1970–1980s. Along with the upcoming of a new era of molecularly-targeted cancer therapy in the early 1990s, the research focus of small-molecule drug discovery, both industry and academia, has shifted to synthesized compounds libraries. This paradigm shift is largely attributed to enormous difficulties encountered in the discovery of effective compounds, the acquisition of sufficient amount of compounds, and the understanding of molecular mechanisms of natural products. Despite the decline of interest, emerging therapeutic concepts and new technologies together cultivate the continuous growth of the field.

### Antibody Drug Conjugates: Old Drugs in New Use

Along with the upcoming of a new era of molecularly-targeted therapy in the early 1990s, the research focus of natural products has shifted to targeted therapies as well. Multiple classes of natural products or their derived drugs are being tested in clinical trials. Among them, a highlight is the antibody drug conjugates (ADCs), which incorporate monoclonal antibody (mAb) and potent cytotoxins in a single molecular entity via chemical linkers. This strategy takes advantage of the targeting capabilities of mAb to enhance tumor-specific drug delivery through the antibody-antigen interaction, thereby sparing normal tissues from cytotoxic effects of traditional chemotherapies [26, 27]. Compelling clinical results with ADCs in both hematological malignancies and solid tumors have prompted renewed interest in the field, which also provides a platform for natural products to nourish.

**Fig. 1:**
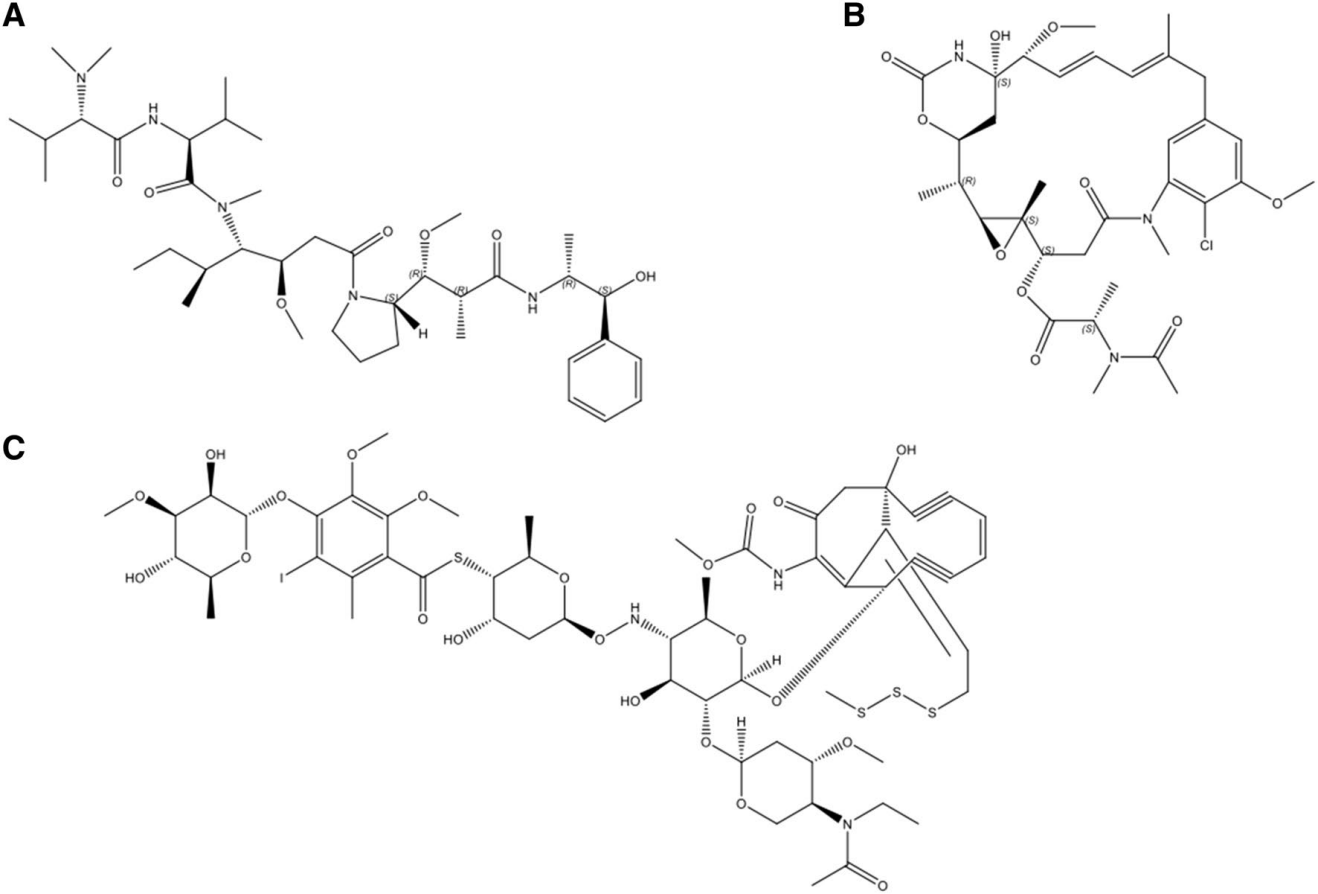
The chemical structures of natural products used in ADCs. **a** Auristatin E, the warhead molecule for MMAE; **b** Maytansine, the warhead molecule for DM1; **c** N‐acetyl‐calicheamicin γ, the warhead molecule for gemtuzumab ozogamicin and inotuzumab ozogamicin

A suitable warhead for ADC development requires certain properties including: (1) a substantially higher toxic potency, with IC50 values below 0.1 nM, compared with most approved chemotherapeutic agents; (2) the appropriate modified site for conjugation with mAb to achieve adequate drug loading; (3) a reasonable solubility in aqueous solutions to enable the reaction with antibodies; and (4) prolonged stability in aqueous formulations commonly used for antibodies [27]. Thus far, the cytotoxic warheads used in ADCs are mostly derived from natural products [1], which mechanistically, can be divided into two main categories: antimitotic agents and DNA-damaging agents. Antimitotic drugs cause cytotoxic effects via disrupting the ability of mitotic spindles to segregate chromosomes or altering the cytoskeleton of cells. The two most widely used antimitotic agents for ADC development are based on auristatins or maytansinoids, whose derivatives, like MMAE and DM1, have been successfully used in clinically approved ADCs (Fig. 1). DNA damaging cytotoxic agents are another class of toxins frequently explored in ADCs. N‐acetyl‐calicheamicin γ is common in ADCs, being used for gemtuzumab ozogamicin and inotuzumab ozogamicin. Other cytotoxic warhead molecules being investigated include derivatives of camptothecins, pyrrolbenzodiazepines, doxorubicin, and centanamycin, duocarmycins, etc.

Gemtuzumab ozogamicin, a conjugate of humanized anti-CD33 monoclonal antibody covalently attached to the cytotoxic antitumor antibiotic calicheamicin, was firstly approved in 2000 under accelerated approval for treating CD33-positive acute myeloid leukemia (AML). The drug was withdrawn from the market in 2010 for failing to demonstrate clinical benefit in a confirmatory post approval clinical trial. Since 2011, nine ADCs have been approved for cancer therapy, with more than 60 ADCs in clinical trials [28]. Brentuximab vedotin was approved in 2011 for treating patients with Hodgkin lymphoma and anaplastic large cell lymphoma. It is a conjugate of anti-CD30 and MMAE, a naturally-derived antimicrotubule agent. Ado-trastuzumab emtansine (T-DM1) was the first ADC approved for treating solid tumors. It was approved by FDA in 2013 to treat HER2 positive (HER2 protein overexpression or HER2 gene amplification) metastatic breast cancer who previously received neoadjuvant taxane and trastuzumab-based treatment. It combines anti-HER2 monoclonal antibody trastuzumab and naturally-derived antimicrotubule agent DM1 via a stable thioether linker, thereby allowing the selective delivery into HER2 positive cells. Very recently, FDA approved sacituzumab govitecan-hziy (IMMU-132), a conjugate of an antibody targeting Trop-2 and the camptothecin derivative SN-38, as the first ADC for treating triple-negative breast cancer.

### Molecularly-Targeted Drug Hunting in Natural Products

The emergence of molecularly-targeted therapies has reshaped the landscape of cancer treatment. As the way the field develops, the interest of natural products research has been drawn to into molecularly targeted drug hunting. These efforts yielded a large collection of natural products with potential activities against various anticancer targets, which, though mostly being immature as a drug candidate, provides diverse chemical scaffolds for drug leads.

**Fig. 2:**
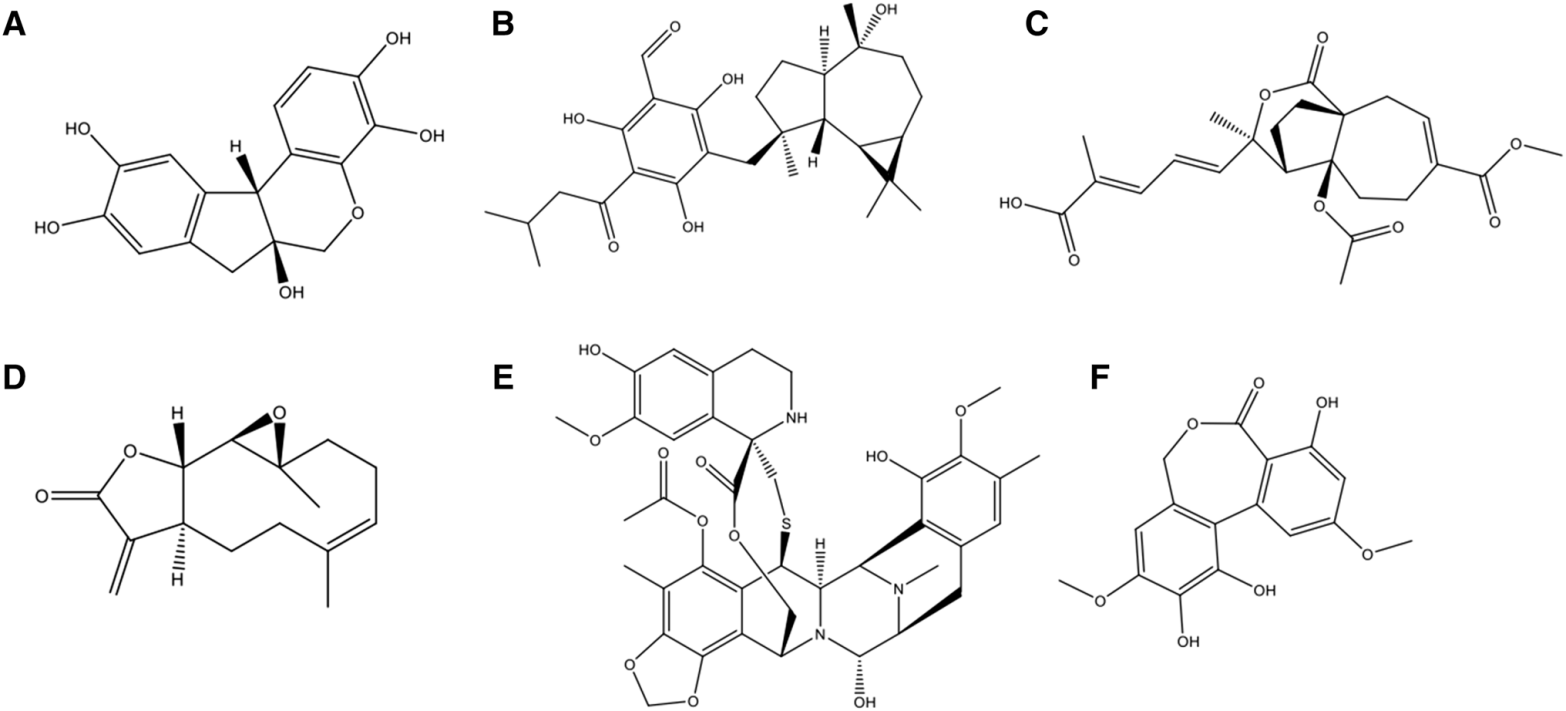
The represent chemical structures of natural products from molecularly-targeted drug hunting. **a** Hematoxylin; **b** Eucalyptin A; **c** Pseudolaric acid B; **d** Parthenolide; **e** Trabectedin; **f** Ulocladol

Among the progress achieved by the natural product research community, Chinese scientists have made their contributions, particularly in plant and marine products. A wide class of botanical bioactive agents has been identified for the activity towards cancer targets (Fig. 2). For example, hematoxylin and its analogues from the heartwood of *Haematoxylon campechianum *were found to be ATP competitive inhibitors of broad-spectrum protein tyrosine kinases, with the highest potency of IC_50_s at nanomolar ranges [29]. Eucalyptin A, which is derived from the fruits of *Eucalyptus globulus *Labill, a plant growing widely in the southwest of China, was found to exhibit potent inhibitory effect on HGF/c-Met axis [30]. Pseudolaric acid B, a diterpenoid isolated from the root bark of *Pseudolarix kaempferi* Gordon tree (Pinaceae), displays anti-angiogenesis activity via a mechanism involving the crosstalk between hypoxia-inducible factor 1-α (HIF-1α) and c-Jun [31, 32]. Parthenolide, a sesquiterpene lactone, firstly purified from the shoots of the medicinal plant feverfew (*Tanacetum parthenium*), showed an inhibitory effect on Wnt/β-catenin signaling that is attributed to its action on ribosome protein RPL10 [33]. Moreover, *Euphorbia peplus* Linn-derived compounds were discovered for the activity to modulate lysosome biogenesis [34]. In addition to botanical drugs, marine natural products are another important source of anti-cancer drug leads, especially those from marine invertebrates. Trabectedin is the first marine-derived anticancer drug originated from Caribbean tunicate *Ecteinascidia turbinate*, yet its mode of action remains unclear [35]. Guo and co-workers discovered several new trabectedin-like dimeric isoquinoline alkaloids and their monomers from marine nudibranchs and their sponge prey, similarly exhibiting significant anti-cancer activities. Further study of these compounds allowed the discovery of the mechanism of NF-κB inhibition, which provides a clue for better understanding trabectedin [36]. Alkaloids apart, marine polyketides could be promising drug leads. For example, a function-oriented synthesis of polyketide ulocladol, which was isolated from marine sponge associated fungi *Ulocladium botrytis*, led to the discovery of a class of inhibitors of M2 isoform of pyruvate kinase (PKM2) [37], a metabolic enzyme critically involved in cancer.

What mentioned here, with no doubt, is only the tip of the iceberg, yet it allows us to get a glance at how the field has evolved gradually from cytotoxic drugs to targeted therapies. With continuous efforts, we may expect more natural compounds reaching the late stage of drug development targeted therapies, and ultimately the clinical use.

### Leveraging Cutting-Edge Technologies to Facilitate the Mechanistic Investigation

Nowadays, the prevailing precision medicines in cancer therapy underscores the necessity to fully understand the molecular basis of anticancer drugs. However, even today, to reveal the direct targets or elucidate the mechanism of natural products remains very challenging. The arising new cutting-edge technologies, such as chemoproteomics and multi-omics, help tackle the obstacles in mechanistic investigation of natural products.

To probe the direct targets of natural products, multiple approaches have been developed. Classical approaches include immobilization of natural products onto solid supports for affinity-based isolation of protein targets [38] and those do not require chemical modification, like cellular thermal shift assay or thermal proteome profiling [39]. Among various approaches, chemoproteomics-enabled strategy has been the dominant one for natural product target identification [40–42]. In this assay, natural products are derivatized to incorporate photoaffinity cross-linkers, biorthogonal handles, and/or biotin enrichment handles to enable covalent capture and enrichment. Activity-based protein profiling (ABPP)-based competitive chemoproteomic profiling is applied to map the proteome-wide targets. For example, nimbolide, an anticancer terpenoid natural product derived from the Neem tree, is found to react with a functional cysteine crucial for substrate recognition in the E3 ubiquitin ligase RNF114 and in turn disrupts RNF114-substrate recognition, leading to inhibition of ubiquitination and degradation of tumor suppressors such as p21 [43]. Likewise, Grossman et al. used chemoproteomic platforms to discover that the anti-cancer natural product withaferin A targets cystine 377 on the regulatory subunit PPP2R1A of the tumor-suppressor protein phosphatase 2A complex, and impaired breast cancer cell proliferation [44].

**Fig. 3:**
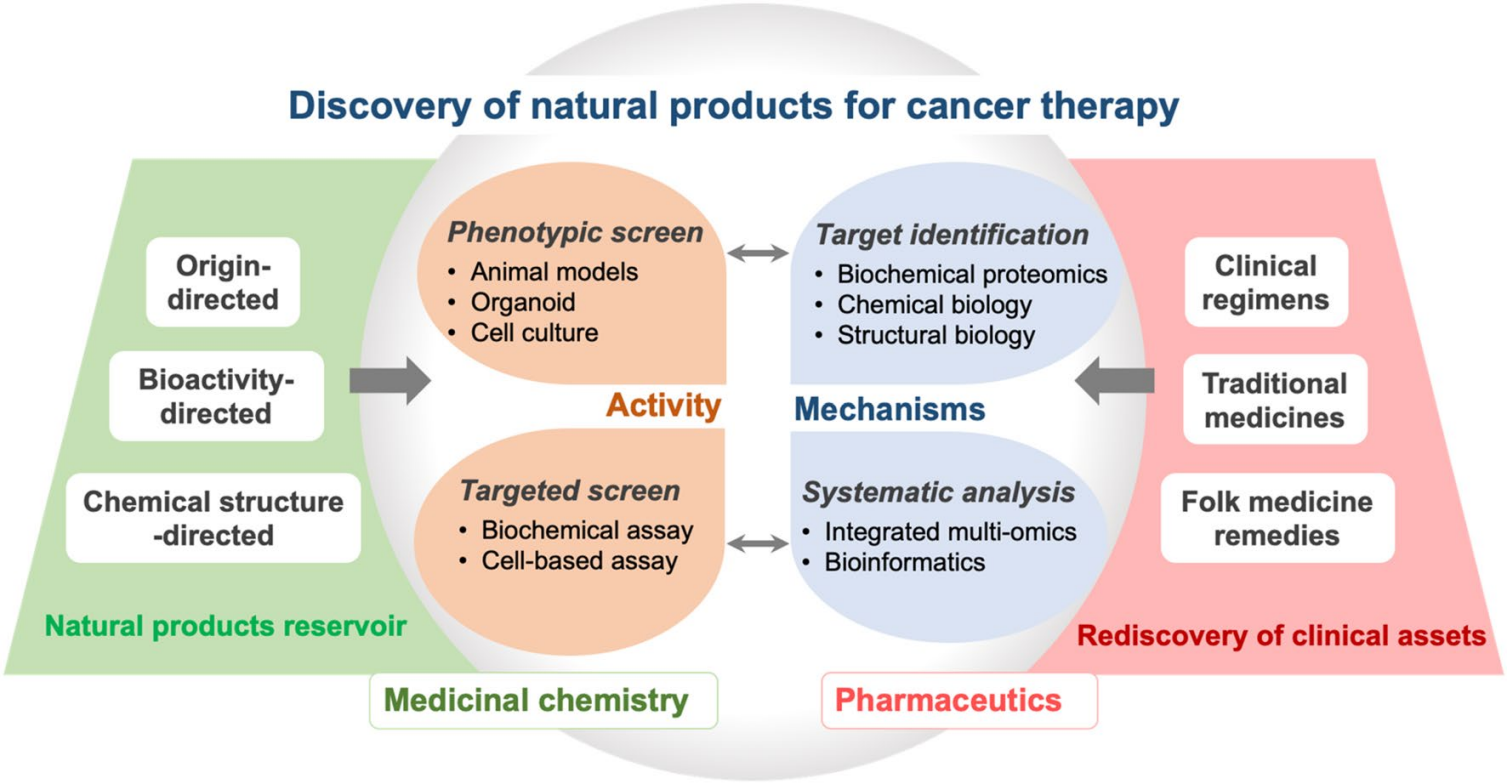
Proposed processes for the discovery of natural products for cancer therapy

In addition to target identification, the growing ability in gaining a comprehensive understanding of cancer-associated molecular alterations has provided an unprecedented opportunity to capture multi-faceted impacts of natural products [45]. In this approach, multi-omic technologies, including genomics, transcriptomics and metabolomics, are systematically characterized and integrated using bioinformatic approaches. This approach will allow revealing molecular pathways and quantified differentially expressed molecules with or without treatment, thereby providing a systematic profile of drug impacts. The integrative approaches in natural products research also promote progress toward the precision medicine paradigm. While most of this kind of study remains at the early stage, limited by phenomenon observation and the lack of in-depth mechanistic investigation, it represents an important direction to conquer the mechanistic complexity of natural products and facilitate drug repositioning (Fig. 3).

## Future Directions that may Open New Opportunities for Natural Products

With remarkable changes in the landscape of cancer therapy and growing role of cutting-edge technologies, we are facing an unprecedent opportunity to better understand nature products and explore their therapeutic utility.

### Integrative Targeted and Phenotypic Screen Accelerates the Discovery of Bioactive Compounds

Up until the 1970s, drug discovery relied heavily on phenotypic screen, an approach aiming to identify active molecules for the desired therapeutic effects regardless of the exact mechanisms behind. In fact, most anticancer natural products were discovered through phenotypic screen [46]. With the growing insights into the molecular alterations in cancer, anticancer drug discovery gradually diverts from phenotypic screen to target-based screen, which is believed to be more efficient and cost-effective to identify drugs with the clarified mechanism of action. When both industry and academia adopt a ‘target-first’ approach for drug hunting, this approach may not be the best to manifest the advantage of natural products in dealing with complex diseases like cancer. Cancer is a heterogeneous disease involving the complex interplay of various internal and environmental factors, with alterations occurring at all levels of DNA, RNA, proteins, metabolites, as well as their interactions [47, 48]. In most cases, inhibition of one single target does not yield optimal therapeutic outcomes or prone to develop resistance even with an initial response. Natural products exhibit an apparent advantage in handling this complexity due to their multi-faceted mechanisms. In this scenario, integrating targeted and phenotypic assays using multi-layers of cancer models (cell lines, organoids and patient-derived xenografts) are expected to better reveal the therapeutic potentials of natural products in cancer therapy. A sophisticated screen platform equipped with natural compound libraries that are differentially classified according to compound origins, chemical structures or bio-activities, will improve the efficiency of drug hunting (Fig. 3).

### Artificial Intelligence Assists the Discovery and Mechanistic Understanding of Bioactive Natural Products

The role of artificial intelligence (AI) in pharmaceutical research is getting increasing attention. A variety of method development efforts and practical applications are being reported, providing a glimpse of how AI technology is entering the drug discovery arena [49]. There is an emerging trend to apply AI approaches to natural products research, in hopes of tackling the challenges in both discovery of bioactive natural products and understanding their mechanisms [50]. Within the scope of natural products research, machine learning algorithms in structure recognition, classification, conformation simulation, library design, and activity prediction are all being actively tested, though mostly at a very beginning stage. Moreover, machine learning-based bioactivity and mechanistic prediction may provide a solution to resolve the complexity of combinations of traditional herbal medicines and plant extracts.

### The Interplay between Host Immune System and Microbiota Opens New Window for Natural Products

Human gut is a biological niche, home to a variety of microbes that affect nearly all aspects of human biology through their interactions with the host. Accumulated evidence supports that the gut microbiome plays pivotal roles in cancer malignancy, via a primary mechanism influencing anti-tumor immunosurveillance [51, 52]. Meanwhile, emerging evidence also supports broad pharmacological effects of natural products on gut microbiota, including the microbiota composition, microbial metabolites, intestinal tight junction structure, and mucosal immunology [53]. In-depth understanding in this regard may resolve a long puzzle of why most natural products exhibit concrete pharmacological effects despite the very limited plasma and tumor exposure. This direction is particularly inspiring for bioactive products derived from TCM as these compounds have century-long experiences with oral administration, and are expected to have a better chance to interact with the gut microbiome.

**Fig. 4:**
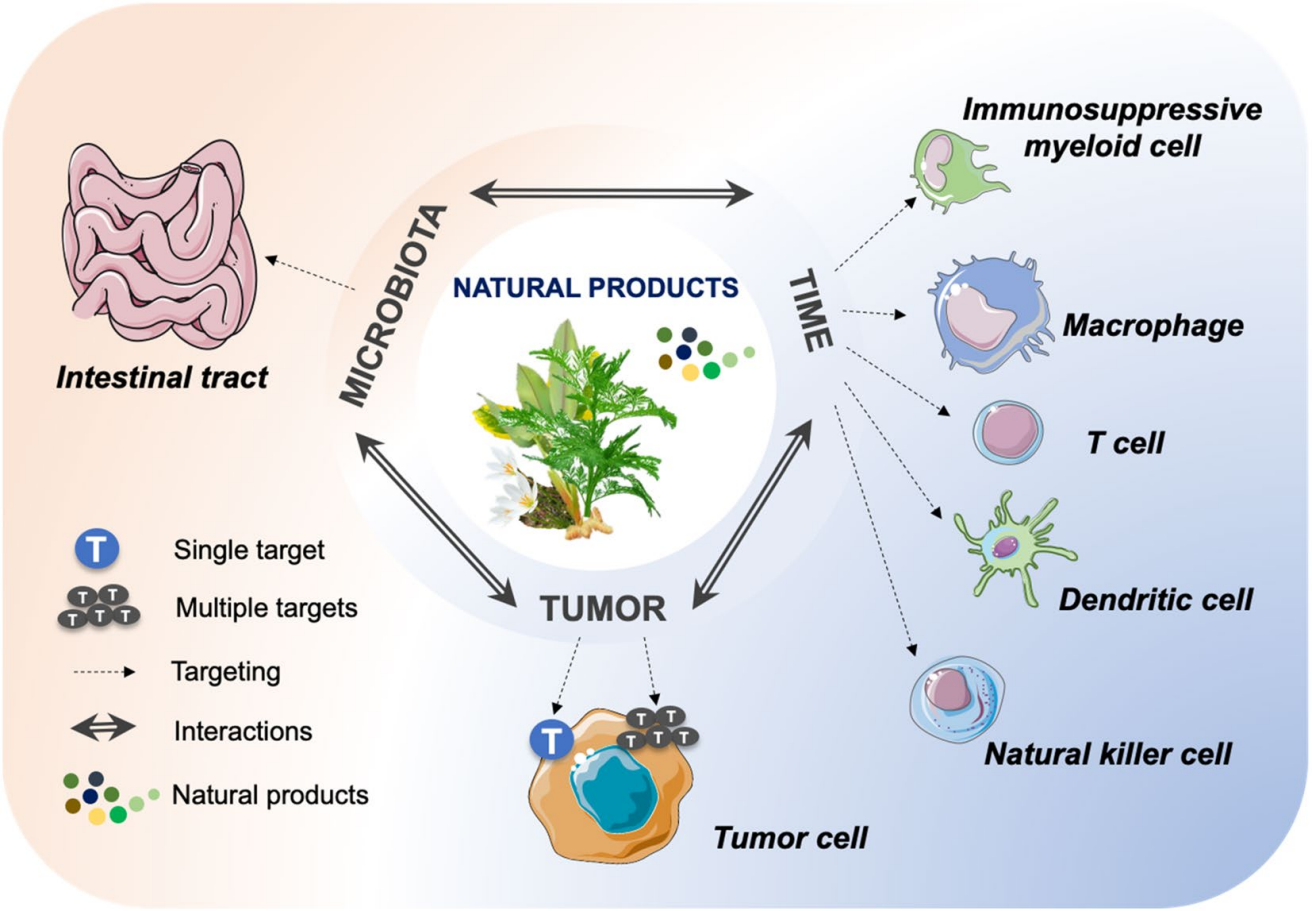
Mode of action of anticancer effect of natural products

Certainly, natural products could also regulate the tumor microenvironment (TME) in a microbiota independent manner. Emerging data suggest the impact of natural products, such as compound kushen injection [54] and icaritin [55], on reshaping the TME via relieving tumor-associated macrophage or reducing the infiltration of myeloid-derived suppression cells. With the compelling success of cancer immunotherapy in clinical treatment, to carefully profile the effects of natural products on the immune cells in TME or the interplay between host immune system and microbiota will open a new window to explore the therapeutic value of natural products and to understand the mechanisms behind (Fig. 4).

### Combination Therapy Broadens the Therapeutic Scope for Natural Products

Combination strategy serves as an important direction for the development of natural products in cancer therapy. Adjuvant treatment of natural products with current regimens could be beneficial in multiple aspects, including reducing the adverse effect, overcoming the drug resistance and improving the therapeutic response. For example, PHY906, a four-herb Chinese medicine formula with century-long history, is reported to alleviate chemotherapy-mediated gastrointestinal toxicity via multiple actions, in stimulating gut cell regeneration, blocking inflammatory cells migration and affecting proinflammatory transcription factors [56]. This work provides a representative model showing how to broaden the therapeutic scope for natural products to optimize the therapeutic effect of cancer therapy. Moreover, the combination therapy strategy is particularly meaningful to bioactive products derived from TCM, which are often limited by the quite modest therapeutic effects in current research models, yet is privileged with decade-long clinical experience.

## Conclusion

Taken together, the past paradigms have evolved to carry on a new role for natural products in the pharmaceutical industry. There are multidimensional problems to be tackled to increase the speed and success rate of drug discovery of natural products. (1) How to select a suitable model to fully reveal the anticancer potential of nature products? Given the heterogeneity of cancer, it has been well-accepted that one compound failing to show activity towards one specific model is not necessarily inactive to other tumor models. Moreover, the anticancer effect of nature compounds could stem from its impacts on the tumor microenvironment or even whole human body. It adds the complexity to select a proper model to manifest the therapeutic potential of candidate compounds. (2) How to efficiently identify the direct targets and the mechanisms of action? Precise cancer treatment will require the full understanding of the mechanism of action of natural compounds. Currently available approaches are still technically quite demanding and mostly in low efficiency. Moreover, as it is quite common that nature products exhibit multi-faceted mechanisms, how to get the whole picture of the mechanism of natural compounds needs to be addressed as well. (3) How to accelerate the process to develop a promising candidate compound to a marketed drug? Most bioactive nature products are facing the issues of large-scale production to meet the manufacturing needs, which constitutes a major hurdle for those promising candidates eventually reaching the bedside. The continuous conceptual advancement in cancer therapy and the implementation of innovative technologies will be needed to resovle these issues and reinforce the historic transformation of the whole field, leading to more clinical success.
